# Respiratory Exposure to Microplastics in Captive-Held Mammals: Validated Lung Tissue Method with Recovery Efficiency and Internal Burden Evaluation in Ungulates

**DOI:** 10.3390/polym18212786

**Published:** 2026-11-07

**Authors:** Laura Cortés Corrales, Sonia Domínguez Tejedor, Harshit Sahai, Mengyan Huang, María Jesús Martínez Bueno, María Dolores Hernando, Amadeo R. Fernández-Alba

**Affiliations:** 1 Experimental Station of Arid Zones, Spanish National Research Council (EEZA-CSIC), Road Sacramento s/n, La Cañada de San Urbano, 04120 Almería, Spain; lcortes@eeza.csic.es (L.C.C.); sdominguez@eeza.csic.es (S.D.T.); mhuang@eeza.csic.es (M.H.); hernando.dolores@eeza.csic.es (M.D.H.); 2 Associated Unit “Chemistry and Environment” of the University of Almería (UAL) and the Spanish National Research Council (CSIC)— Experimental Station of Arid Zones (EEZA), E-04120 Almeria, Spain; mjbueno@ual.es; 3 Department of Chemistry and Physics, Research Centre for Mediterranean Intensive Agrosystems and Agri-Food, Biotechnology (CIAIMBITAL), University of Almeria, Agrifood Campus of International, Excellence, ceiA3, E-04120 Almería, Spain; harshitsahai@ual.es

**Keywords:** microplastics, lung, captive-held ungulates, gazelle, whole-organ analysis, recovery efficiency, µFTIR, inhalation exposure, bioindicator, prenatal transfer

## Abstract

Respiratory exposure to microplastics (MPs) remains poorly quantified because most tissue-based studies neither report extraction recovery efficiency nor assess whole-organ burden. Using lamb lungs as a model, we validated a method for determining MP burden in lung tissue by comparing three digestion oxidants and three sample masses against four spiked reference polymers:polypropylene(PP),polystyrene (PS),low-density polyethylene (LDPE) and polyethylene terephthalate (PET). Sodium persulfate (Na_2_S_2_O_8_) digestion of 10 g freeze-dried tissue (~25% of the lamb lung) gave the best recovery (~70%) and was applied to lung tissue from captive-held Saharan ungulates (two adult gazelles, a pregnant gazelle with two fetuses, and a neonatal aoudad). Across the adult gazelles, fibres dominated the internal burden, with PP enriched among fragments and PET dominant among fibres, confirmed by micro-Fourier- transform infrared spectroscopy (µFTIR). Particles were overwhelmingly coarse (mostly 30 to ≥100 µm), with essentially none meeting respirable (<10 µm) or alveolar-like (<4 µm) size criteria, consistent with retention in conducting airways rather than alveolar deposition. No MPs were detected in either fetus or in the neonatal aoudad, suggesting postnatal rather than prenatal accumulation of pulmonary burden. This is, to our knowledge, the first recovery-characterized, whole-lung (one animal) and per-gram tissue-level (two animals) burdens in captive-held ungulates, providing a transferable framework for assessing respiratory MP exposure in captive-held life and livestock.

## 1. Introduction

Global plastic production and associated waste generation have continued to increase over recent decades, with forecasts pointing to a possible tripling of both plastic production and plastic waste by the middle of this century [1,2]. This growing pressure is driving the continuous fragmentation of plastic materials in the environment, giving rise to microplastics (MPs, <5 mm) and nanoplastics (NPs, <1 µm), which have now been detected in virtually all environmental compartments and in a wide range of organisms [2,3,4]. Initially, scientific attention focused on dietary exposure, but in recent years evidence has accumulated demonstrating the presence of MPs in human tissues such as placenta, lung, blood, and breast milk, confirming that exposure is systemic and probably lifelong [3,4,5,6,7].

In the field of food safety, numerous studies have demonstrated the presence of MPs both of environmental origin (foodstuffs that accumulate MPs during production) and directly released from food-contact materials (packaging, bottles, disposable cups, infusion bags, etc.) [2,8,9]. However, comparison between studies reveals discrepancies of several orders of magnitude in the reported concentrations for the same matrix, such as table salt or bottled water. Canga et al. showed that these differences are largely explained by the absence of standardized protocols and by the enormous variability in analytical strategies employed, which compromises the comparability of results and the realistic estimation of exposure to MPs through foods and beverages [8]. This lack of harmonization echoes earlier concerns raised by international bodies such as WHO, which have emphasized that uncertainty in exposure evaluation is driven at least as much by methodological limitations as by genuine environmental variability [2,9].

In parallel, respiratory exposure to MPs has emerged as an area of growing interest, although it remains comparatively understudied. Various reviews and monitoring studies have demonstrated the presence of plastic particles in outdoor air and, very prominently, in indoor environments, where fibres from synthetic textiles, household dust, and the wear of plastic materials can represent a substantial fraction of the inhaled burden [10,11,12]. Recent data indicate that airborne MPs and NPs are now recognized components of particulate matter, capable of long-range transport and deposition [1,11]. MPs have been identified in human lung tissue, particularly in individuals with occupational exposure to synthetic fibres and dust, and there is increasing concern that particle deposition in the alveolar region may trigger chronic inflammatory processes analogous to those described for fine particulate matter (PM2.5), with potential links to asthma, chronic obstructive pulmonary disease (COPD), pulmonary fibrosis, and lung cancer [5,12,13,14,15,16]. Despite this, neither recent WHO reports on MPs nor most air-quality regulatory frameworks explicitly include respirable MPs within the particulate matter fractions of regulatory interest, highlighting a clear misalignment between emerging scientific concerns and the current regulatory response [1,2,9,11].

Several authors have pointed out that the cornerstone for advancing microplastic risk evaluation is no longer to demonstrate their presence—already extensively documented—but rather to establish validated quantitative methods capable of reliably estimating internal burdens (particle number, size distribution and polymer composition) in different biological matrices [3,8,17,18]. This need is particularly acute when working with complex biological tissues, such as animal or human organs. In such cases, MP analysis is especially prone to bias arising from multiple sources: contamination during sampling and handling, non-representative tissue selection, incomplete or overly aggressive digestions that may destroy specific polymers, filtration steps with size-dependent efficiencies, and misidentification of particles when spectroscopic techniques, such as micro-Fourier- transform infrared spectroscopy (µFTIR) or micro-Raman (µ-Raman) are not systematically applied to confirm their polymeric nature [3,17,18].

The absence of systematic recovery measurements and of a clear evaluation of sample representativeness is a critical limitation that hampers the establishment of any toxicologically meaningful dose–response relationship and prevents robust risk evaluation [8,17,18]. Recent methodological studies have begun to address these shortcomings through review-based good-practice frameworks for sampling, contamination control and data reporting in MP research, as well as by proposing approaches for recovery evaluation and quality control in microplastic analysis [17,18]. However, to the best of our knowledge, none of the studies reporting the presence of microplastics in animal tissues (e.g., lung in wild birds, penguins, or in mussels and canned mackerel [19,20,21,22]) or in human tissues (placenta, lung, colon, heart, among others [4,5,23,24,25]) have described the recovery efficiency of the extraction method used to isolate MPs from the sampled tissue at the level of a complete organ. Even the very few, very recent studies that do report a formal recovery efficiency for microplastic extraction from an animal tissue matrix have applied it to small gastrointestinal tissue subsamples or homogenized soft tissue, not to a complete organ from a wild mammal [26,27,28]. Nor has the internal MP burden been characterized at the whole-organ level; to date, studies rely on small tissue subsamples, often poorly representative of the entire organ (in terms of size or location), and in most cases the results are reported as particles per gram of tissue. To our knowledge, no previous study has combined a validated recovery efficiency with organ-level (whole-lung) internal burden estimation in any captive-held mammal. This lack of quantitative recovery data and organ-specific burden metrics further limits the evaluation of dose–response relationships of toxicological relevance and hinders any solid evaluation of the potential impact of MPs on animal or human health.

Given the inherent difficulties of MP analysis in biological tissues—owing to matrix complexity, high lipid and protein content, and the elevated risk of contamination—it is essential to develop methods that combine sufficiently effective chemical digestion to remove organic matter with separation and filtration procedures that preserve the integrity of plastic particles, together with spectroscopic identification techniques capable of discriminating MPs from other inorganic or organic fragments [3,5,17,18]. Such methods need exhaustive validation, including the evaluation of recovery by spiking with MPs to determine internal burdens in specific organs, the systematic use of field and laboratory blanks, and the implementation of quality-control measures throughout the analytical process [17,18].

In this context, the aim of the present study was to develop and validate an analytical procedure for the determination of MPs in animal lung tissue, using lamb lung as a model. The method was designed with particular emphasis on evaluating the recovery efficiency of the MP extraction procedure from the sampled tissue, assessing the internal MP burden at the organ level (lung), and implementing stringent quality control throughout the analytical process, in order to ensure its reliability and enable its future application in studies of MP detection in other mammals. Inhalation of MPs may also constitute a relevant route of exposure for ungulates, especially in arid and semi-arid regions where wind regimes and sparse vegetation cover favour the atmospheric transport and resuspension of dust [10,11,13]. Under these conditions, animals may be chronically exposed to substantial levels of dust and suspended particles, with potential consequences for their respiratory health. Moreover, such studies in mammals are particularly relevant because of their potential usefulness as bioindicators of MP contamination in terrestrial ecosystems [12,13,29,30,31,32]. The study is based on samples obtained from necropsies of different species of captive Saharan wild ungulates, including adults, fetuses and neonates, thereby opening up the possibility of exploring not only chronic respiratory exposure but also potential prenatal transfer of MPs [4,7,33,34]. The results derived from this work aim to provide a solid methodological basis for the quantification of MPs in lung tissue and to contribute to our understanding of respiratory exposure to these particles in mammals, with implications for both ecology and public health [1,2,3,11,12,13].

## 2. Materials and Methods

### 2.1. Quality Assurance and Quality Control (QA/QC)

A quality assurance and quality control (QA/QC) protocol was implemented to ensure the accuracy and reliability of the final data. All sample preparation was carried out in a clean-air environment under a laminar-flow cabinet equipped with air purification systems to minimize cross-contamination. Laboratory personnel wore non-synthetic clothing (cotton lab coats) to reduce the shedding of synthetic fibres. All materials used during sample treatment and analysis (glassware, stainless-steel tools, filtration devices, jars and Petri dishes) were pre-cleaned by sequential rinsing with filtered Milli-Q water (Central Research Service of the University of Almeria, Spain), acetone and methanol (Honeywell Specialty Chemicals, Seelze, Germany) (0.2 µm), followed by heating in an oven at 200 °C for 6 h. When not in use, all containers and equipment were covered with aluminium foil. One procedural blank was processed in parallel with the samples of each animal and underwent all steps of the procedure, including digestion, density separation, filtration and µFTIR analysis. For Dorcas 2, a single procedural blank covered all five subsamples. Four additional blanks were processed during the validation experiments under the same conditions as the validation tests, with one blank for each of the four solvent tests. Particles identified as cellulose, rayon or cotton were excluded from the counts in blanks and samples alike, as they could not be confidently assigned to synthetic polymers. No synthetic polymer particles were detected in the procedural blanks; therefore, no blank correction was applied.

### 2.2. Sample Collection and Pre-Treatment

Fresh lung tissue was obtained from a local butcher shop by purchasing three lamb lungs of approximately 200 g each. The lungs were rinsed with filtered Milli-Q water, placed in pre-cleaned glass jars, carefully sealed, and stored at −20 °C until homogenization. Sample homogenization followed a freeze-drying and milling procedure to maximize representativeness and ensure the reliability of the developed method. Frozen lungs were thawed at room temperature, and using stainless-steel scissors and tweezers, the tissue was cut into small pieces and distributed onto plastic Petri dishes that had been rinsed (five times) with 0.2 µm-filtered Milli-Q water. At this stage, only plastic Petri dishes were selected, rather than glass, to prevent breakage during freeze-drying. The Petri dishes were then placed in a Telstar freeze-dryer (Telstar, Terrassa, Spain). The process consisted of two stages: (i) freezing at −50 °C for 24 h and (ii) water removal (primary drying) for 48 h. After completion of freeze-drying, the dried tissue was milled in a Retsch MM 400 ball mill (Retsch GmbH, Haan, Germany) for 5 min at 5000 rpm. The resulting powder was transferred using a stainless-steel spatula into pre-cleaned glass jars (see QA/QC cleaning procedure above), which were stored at −20 °C until further use. Approximately 40 g of freeze-dried lung was obtained from each ~200 g fresh lung, indicating a water content of around 80% in lamb lung tissue.

### 2.3. Sample Representativeness

Method validation was carried out using different amounts of freeze-dried lung tissue to assess method performance as a function of sample mass. Subsamples of 2 g, 10 g and 20 g were tested to determine both sample representativeness and the maximum amount of tissue that could be processed without compromising precision and accuracy. For each mass level, representativeness relative to the original fresh lung was calculated: a 2 g subsample of freeze-dried lung corresponded to approximately 5% of the fresh lung mass, 10 g to 25%, and 20 g to 50%.

### 2.4. Recovery Experiments

After sample preparation and homogenization, recovery experiments were carried out by fortifying the freeze-dried lung tissue with known numbers of MPs (40 particles, 10 particles for each type of polymer studied). This step is critical in method development, as it allows evaluation of method trueness, a key parameter for assessing method validity. Four polymer types were used for spiking: polypropylene (PP), polystyrene (PS), low-density polyethylene (LDPE) and polyethylene terephthalate (PET). PP MPs consisted of white fragments < 50 µm, PS MPs were also white fragments but in the 50–200 µm range, LDPE MPs were black fragments < 50 µm, and PET MPs were orange fibres < 50 µm. For each mass level (2, 10 and 20 g), freeze-dried tissue stored at −20 °C was allowed to reach room temperature before weighing. Aliquots were weighed on an analytical balance, inside a laminar-flow hood, using pre-cleaned aluminium foil as weighing boats. The required amount of tissue was transferred with a stainless-steel spatula into pre-tared 250 mL glass jars with bayonet-type caps (Labbox Labware, S.L., Premià de Dalt, Spain). Spiking was carried out under a Leica S9i stereomicroscope (Leica Microsystems, Wetzlar, Germany). Individual MPs were picked up with a fine needle and placed onto a glass sample holder. Once the desired number and combination of MPs had been selected, the glass holder was carefully introduced vertically into the glass jar and the jar was immediately closed. The same procedure was applied for 2 g, 10 g and 20 g samples, with five replicates prepared per mass level. Total recovery for each replicate was calculated as the percentage of the 40 spiked particles recovered. Differences in total recovery among the three oxidants (2 g of tissue) were tested by one-way analysis of variance (ANOVA) (Microsoft Excel for Microsoft 365, version 2610).

### 2.5. Digestion Step

Following spiking, samples were subjected to chemical digestion to remove organic matter. To optimize the digestion conditions, three oxidizing reagents were evaluated: 10% (*w*/*v*) potassium hydroxide (KOH), Fenton’s reagent and sodium persulfate (Na_2_S_2_O_8_)solution (40 g/L). In addition, a control experiment using only a saturated calcium chloride (CaCl_2_) solution (750 g/L) without oxidant was performed to assess the necessity of including an oxidative digestion step. Fenton’s reagent was prepared by mixing hydrogen peroxide (H_2_O_2_; 30%), iron (II) sulfate heptahydrate (FeSO_4_· 7H_2_O; 2 mmol/L), sulfuric acid (H_2_SO_4_; 2 mmol/L) and Milli-Q water in a 10:1:1:5 volume ratio. For optimization, five replicates were processed with each oxidizing agent using 2 g of freeze-dried lung per sample. For the 2 g tests, 30 mL of each oxidizing solution was dispensed using a 50 mL glass cylinder and added to the glass jars containing the spiked samples. MPs were transferred from the glass sample holder into the jar using the digestion solution as a rinse to ensure quantitative transfer. Jars were then sealed and agitated for 12 h for the KOH and Fenton’s reagents. For Na_2_S_2_O_8_, samples were placed in an oven at 50 °C for 24 h to thermally activate the persulfate. In this case, the jars were covered with glass watch glasses instead of screw caps to avoid potential degradation of the caps and loss of plastic particles. Based on the results of the optimization experiments, the digestion of 10 g and 20 g samples was carried out exclusively with Na_2_S_2_O_8_ (40 g/L), following the same procedure as for 2 g samples but adjusting the volume of reagent. The volume of digestion reagent was 15 mL/g of dry tissue for 2 g and 6 mL/g for 10 g and 20 g. Five replicates were prepared per condition. Replicates were excluded from the calculations in the following cases: KOH, replicate 1 (a precipitate formed on adding CaCl_2_ and the sample was lost; NaCl was used from replicate 2 onwards); Fenton, replicate 1 (sample lost while recovering the particles); CaCl_2_ only, replicates 3–5 (analysis was not possible because of the amount of residual organic matter and very slow filtration).

### 2.6. Density Separation

After digestion, density separation combined with clarification by centrifugation was used to separate MPs from the residual matrix. Saturated solutions of CaCl_2_ (≈750 g/L) and NaCl were employed. The theoretical density of a saturated CaCl_2_ solution at ≈20 °C is ≈1.40 g/cm^3^, and that of the NaCl solution is 1.20 g/cm^3^; the density of the working solutions was not measured. The density of PET ranges from 1.33 to 1.45 g/cm^3^ depending on crystallinity [35]. Samples were centrifuged for 5 min at 1835× *g*. The densities of the polymers used for fortification were: PS (1.04–1.10 g/cm^3^), PP (0.90–0.91 g/cm^3^), LDPE (0.93 g/cm^3^) and PET (1.37 g/cm^3^). For 2 g samples treated with Fenton’s reagent or Na_2_S_2_O_8_, a saturated CaCl_2_ solution was used. However, for samples treated with 10% KOH, the addition of saturated CaCl_2_ produced a precipitate in the first replicate. This was attributed to the formation of poorly soluble Ca(OH)_2_ when Ca^2+^ was added in excess to a solution containing high concentrations of KOH. Consequently, for the remaining KOH-digested replicates, saturated NaCl solution was used instead of CaCl_2_. In all cases, 20 mL of saturated CaCl_2_ or NaCl solution was measured in a 50 mL glass cylinder and added to the digestion jars, which were then gently shaken. The contents were subsequently transferred to two 50 mL Falcon tubes and centrifuged at 4000 rpm for 5 min. For 10 g and 20 g samples, saturated CaCl_2_ was used in combination with Na_2_S_2_O_8_ digestion, as no precipitation problems were observed. For 10 g samples, 30 mL of CaCl_2_ solution was added, and the suspension was distributed into three 50 mL Falcon tubes. For 20 g samples, 120 mL of CaCl_2_ solution was added, and the suspension was divided among four 50 mL tubes before centrifugation.

### 2.7. Filtration and Mesh Cleaning

After centrifugation, the supernatant containing the MPs was vacuum filtered through a 10 µm stainless-steel or polymer mesh placed in a Büchner funnel. The mesh was carefully removed with stainless-steel tweezers and placed in a pre-cleaned glass jar. A volume of 30 mL of pre-filtered distilled water was added (measured with a 50 mL glass cylinder), and the jar was placed in an ultrasonic bath (Digital Ultrasonic Cleaner 30L; Vevor, Shangai, China) for 20 min to detach MPs from the mesh. After sonication, the mesh was removed with tweezers, and the suspension was filtered through 1.2 µm cellulose ester filters (S-Pak, Merck Millipore, Milford, MA, USA) under vacuum. The filters were then placed in Petri dishes and left to dry at room temperature until analysis. The cleaning protocol for meshes consisted of: (i) 1 h in an ultrasonic bath with filtered dichloromethane (Honeywell Specialty Chemicals, Seelze, Germany), (ii) drying in an oven at 200 °C for 12 h, (iii) 30 min in an ultrasonic bath with filtered methanol, and (iv) a final drying step in the oven at 200 °C for 6 h. This protocol was applied before first use and between batches to minimize carry-over and contamination.

### 2.8. Necropsy of Ungulates and Lung Tissue Sampling

The analytical procedure developed and validated using lamb lung as a model (Section 2.1, Section 2.2, Section 2.3, Section 2.4, Section 2.5, Section 2.6 and Section 2.7) was subsequently applied, without modification, to the lung samples from Saharan ungulates. This guaranteed that the results obtained for captive-held ungulates were fully comparable with those from the controlled recovery experiments in lamb, and that the associated performance characteristics (precision, accuracy and recoveries) were directly transferable to the application dataset. After death, the carcasses were kept frozen in chest freezers at temperatures up to −18 °C until necropsy, which was delayed until a member of the research team could attend to collect the samples (2–4 days after death; Table 1). The carcasses were wrapped in durable polyethylene (PE) plastic bags and allowed to thaw at room temperature. After necropsy, the lungs were placed in aluminium foil bags and stored at −20 °C until homogenization. Lung tissue was collected from captive Saharan ungulates during routine veterinary necropsies. Animals included adults, fetuses and neonates that died from natural causes or medical conditions unrelated to this study. All procedures were carried out by veterinary staff in a dedicated necropsy room and in accordance with institutional guidelines. To minimize microplastic contamination, specific QA/QC measures were implemented in the necropsy room. Only stainless-steel instruments (scalpels, scissors, forceps) and pre-cleaned glass containers or aluminium bags were used for tissue collection. Instruments and containers were rinsed with filtered Milli-Q water and covered with aluminium foil prior to use. Necropsy staff wore cotton gowns and non-shedding footwear; synthetic overalls and disposable plastic aprons were avoided whenever possible. The use of paper towels, plastic sponges and other potential sources of fibres was reduced to a minimum and restricted to areas away from the sampling field. During necropsy, thoracic cavities were opened using standard veterinary procedures, and lungs were exposed with care to avoid contact with external surfaces and clothing. For Cuvier gazelle and Dorcas 1, a representative portion of each lung was obtained, made up of small pieces taken from different parts of each lobe; for Dorcas 2, the whole lung was excised; for fetus and neonates, the entire lungs were removed. Each lung (or lobe) selected for analysis was immediately placed in a pre-cleaned glass jar, sealed with a metal cap and covered with aluminium foil, or placed in an aluminium bag. Samples were labelled, transported to the laboratory in insulated containers, following the procedure described in Section 2.1 and Section 2.2. The lungs were rinsed with filtered Milli-Q water, placed in pre-cleaned glass jars, carefully sealed, and stored at −20 °C until homogenization.

This protocol ensured that lung tissue samples from ungulates were collected and handled under controlled conditions suitable for trace-level microplastic analysis. Lung samples were obtained from two adult Dorcas gazelles (*Gazella dorcas*), one Cuvier’s gazelle (*Gazella cuvieri*) carrying two fetuses (one female and one male) that died before birth, and one 5-day-old aoudad (*Ammotragus lervia*). For Cuvier gazelle and Dorcas 1, the portions weighed 108 g and 76 g fresh and were freeze-dried and analyzed in full (21 g and 15 g dry; water content ≈ 80%). The total weights of these lungs are unknown. The lung of Dorcas 2 (249 g fresh, ≈50 g dry) was analyzed whole, as five replicates of 10 g. Specific information for each individual (species, sex, age class and sample type) is summarized in Table 1. As no synthetic particles were detected in any procedural blank, a statistical detection limit based on blank variability could not be defined. The detection limit was therefore expressed as one particle per gram of dry tissue processed, corrected for the mean recovery obtained for 10 g of tissue (0.69): detection limit = 1/(m × 0.69), where m is the dry mass processed (g).

Animals were kept in outdoor enclosures at Hoya Experimental Farm in Almería (southeastern Spain). Fences, shelters and feeders were made of metal, and the ground was sand and, in some areas, concrete. The only plastic items inside the enclosure were the holders supporting the salt licks and the shade cloth fitted on the sunshades. Windblown plastic bags occasionally entered the premises and were removed as soon as detected.

### 2.9. Microscopic and µFTIR Analysis

An important analytical consideration for interpreting the size-based results presented later in this study concerns the intrinsic detection capability of transmission µFTIR at the lower end of the microplastic size range. Because full-range spectral acquisition (4000–650 cm^−1^) relies on mid-infrared radiation with wavelengths of approximately 2.5–15 µm, the spatial resolution attainable by conventional transmission µFTIR is fundamentally diffraction-limited to a comparable order of magnitude. In practice, obtaining a spectrum with sufficient signal-to-noise and spectral definition to exceed the 65% library-match threshold (see below) becomes increasingly difficult for particles approaching, or smaller than, ca. 4 µm, which coincides with the operational “alveolar-like” size cut-off adopted for particle classification in Section 3.2. Consequently, particles at or below this size are more likely to be missed, mis-segmented, or excluded for failing the identification threshold than to be confidently confirmed as microplastics, and the present method should be regarded as conservative—rather than exhaustive—for this smallest size fraction; where sub-4 µm coverage is a priority, complementary techniques offering finer spatial resolution (e.g., µRaman spectroscopy) are recommended [3,17,18].

After extraction, MPs retained on the cellulose nitrate filters were first examined using a Leica S9i stereomicroscope equipped with a 10 megapixel digital camera. MPs were transferred manually, one by one, from the 1.2 μm cellulose nitrate filters and deposited on the surface of microporous silicon membranes MakroPor P12M5-500 (Smart-Membranes GmbH, Halle, Germany; pore diameter 5.5 μm, inter-pore distance 12 μm, thickness 475 μm, size 10 mm × 10 mm) under the stereomicroscope, using a fine needle, and all the particles were analyzed by Fourier-transform infrared (FTIR) microspectroscopy in semi-automatic mapping mode (mapping of the area of the membrane carrying the particles). The lower size limit of the method is set by the visual detection and manual handling of the particles and by the spatial resolution of the μFTIR, not by the pore diameter; the smallest particle identified was a fragment 15.9 μm long, so particles of a few micrometres, including those below the ~4 μm threshold discussed in Section 4.3, were not covered. A Nicolet iN10 micro-FTIR (Thermo Fisher Scientific, Waltham, MA, USA) was used to acquire transmission spectra in the 4000–650 cm^−1^ range, with a spectral resolution of 8 cm^−1^ and 16 scans per spectrum. To improve sensitivity and reduce background noise, the internal mercury cadmium telluride (MCT) detector was cooled with liquid nitrogen. A daily calibration routine was carried out before analysis to correct for atmospheric CO_2_ and water vapour interferences. Recorded spectra were carefully inspected and compared with polymer spectral libraries supplied by Thermo Fisher Scientific, as well as with in-house reference spectra. A match threshold of 65% spectral similarity was set as the minimum criterion for positive identification of a polymer type, as this level was considered to indicate sufficiently representative agreement with reference spectra. All spectra exceeded this threshold, so no spectrum was discarded. All the particles selected under the stereomicroscope were transferred and analyzed by μFTIR; very small, transparent or white particles may have gone unnoticed during the visual selection (the smallest particle identified was a fragment 15.9 µm long). This rigorous analytical workflow ensured reliable chemical and morphological characterization of the MPs isolated from lung tissue. Particles identified as cellulose, rayon or cotton were not considered synthetic microplastics and were excluded from the counts, in samples and procedural blanks alike.

Particles were classified by their morphology as fibres (elongated, thread-like particles of roughly constant width), fragments (irregular or angular particles without a thread-like shape) or films (thin, flat, translucent particles), by visual inspection of the stereomicroscope and image analysis. The aspect ratio did not separate the classes by itself (median 4.9 in fibres and 1.7 in fragments) and was recorded as a descriptor, not used as a criterion. Dimensions were measured with the Leica Application Suite X (LAS X 3.9). Particle outlines were segmented to obtain contour-based morphometric descriptors, including projected area (µm^2^), maximum length (µm; major axis), mean width (µm; minor-axis proxy), and, for fibres, centreline (skeleton) curve length (µm). Additional descriptors were extracted to capture non-sphericity and elongation, including aspect ratio (length/width), the equivalent circular diameter (ECD, µm; diameter of a circle with the same projected area), Feret 0 (µm; horizontal Feret diameter), roundness (dimensionless; 1 for a perfect circle), and compactness/irregularity metrics (e.g., fullness ratio, shape factor, curve ratio). All descriptors were exported to spreadsheets for downstream processing.

To provide a consistent size descriptor across irregular fragments and elongated fibres, a geometric diameter proxy (d_g) was defined using the available 2D metrics. For fragments, d_g was set equal to ECD (d_g = ECD), representing an area-equivalent projected diameter derived directly from the measured contour. For fibres, where penetration is primarily governed by the critical dimension (width) but projected area also contains information on thickness and irregularity, d_g was defined as a conservative combined proxy incorporating both width and ECD (d_g = √(Width × ECD), i.e., the geometric mean of width and ECD). This hybrid definition intentionally penalizes thicker fibres and increases sensitivity to both cross-sectional and area-derived size, while remaining computable for all fibres in the dataset.

Because a true aerodynamic diameter (d_a) requires information not directly recoverable from 2D microscopy (e.g., particle density at the individual level, dynamic shape factor, and orientation-dependent drag), an aerodynamic diameter sensitivity proxy (d_a,proxy) was computed for comparative purposes rather than as an absolute aerodynamic measurement. The proxy was calculated as d_a,proxy = d_g × √ρ_p × √χ, where ρ_p is an assumed polymer density (g·cm^−3^), and χ is a dimensionless shape correction derived from morphometric descriptors that reflect elongation and departure from circularity. Polymer density was assigned deterministically at the particle level according to the polymer type identified for that particle by µFTIR against reference spectral libraries (Section 2.9), using typical literature densities for each identified polymer (PP 0.90, PE 0.92, PET 1.37, PS 1.05, and PA/polyamide ≈ 1.14 g·cm^−3^). The shape correction χ was parameterized as an increasing function of elongation (aspect ratio) and non-circularity (1—roundness), with optional modulation by compactness/irregularity descriptors (e.g., fullness ratio, shape factor, curve ratio) when available, so that more elongated and/or irregular particles yield larger χ and thus a higher d_a,proxy relative to d_g. To reflect uncertainty associated with orientation and the 2D-to-3D mapping of drag behaviour, sensitivity bounds for d_a,proxy (low/high) were additionally reported by propagating conservative lower and upper limits for χ.

To support interpretation of potential penetration into the lower respiratory tract, particles were operationally classified using commonly applied aerosol cut-offs, treating these categories as comparative descriptors rather than species-specific deposition guarantees: “respirable-like” (<10 µm) and “alveolar-like” (<4 µm). These thresholds were applied in parallel using (i) d_g as a purely geometric screening criterion and (ii) d_a,proxy as a sensitivity analysis incorporating density and shape. For each animal and lung region (lobe), summary statistics (*n*, median, and 90th percentile) of d_g and d_a,proxy were calculated separately for fibres and fragments, together with the percentage of particles below 10 µm and 4 µm under each criterion, and compiled into the manuscript table. All computations and table generation were performed in Microsoft Excel for Microsoft 365, version 2610, and only objects with unambiguous segmentation (closed contour, consistent morphology, and valid metric output) were retained for analysis. To minimize artefactual contamination, sample handling and imaging were conducted under contamination-controlled conditions, supported by procedural blanks and workflow controls.

## 3. Results

### 3.1. Recovery Efficiency and Organ-Level Internal Burden Evaluation: Method Validation

Freeze-dried lung subsamples of 2, 10 and 20 g were subjected to digestion with three oxidants—Fenton’s reagent, 10% KOH and Na_2_S_2_O_8_—as described in the experimental section. Saturated CaCl_2_ was used for density separation, except in the KOH treatment, where NaCl was used from the second replicate onwards because CaCl_2_ produced precipitate; saturated CaCl_2_ without any oxidant provided a benchmark condition. Five replicates were prepared per condition; the number of replicates finally analysed, together with polymer-specific and total recoveries, is a given in Table 2.

For 2 g subsamples, the choice of oxidizing agent had a marked impact on both matrix removal and recovery. When no oxidant was used and only saturated CaCl_2_ was added, a large amount of undigested organic matter remained after 24 h, leading to heavily loaded, dark filters that made particle counting difficult and increased the risk of misclassification. Under these conditions, only two of the five replicates could be analyzed, and the mean recovery was about 30% (30.0 ± 10.6%). The use of Fenton’s reagent improved matrix digestion, but only partially: filters still showed visible residues that hindered visual inspection and microplastics characterization. Nonetheless, the average recovery increased to 69.4 ± 3.8% (*n* = 4). Digestion with 10% KOH further enhanced matrix degradation, generating cleaner filters with lower organic loading and simplifying microscopic examination; the mean recovery achieved with KOH (63.8 ± 4.3%, *n* = 4; NaCl was used for density separation in this treatment) was slightly below that obtained with Na_2_S_2_O_8_, which removed organic matter more efficiently, yielding clean filters with minimal residual material and greatly facilitating both filtration and MP isolation. Its mean recovery was 70.5 ± 4.1% (*n* = 5), and the recoveries of the three oxidants did not differ significantly (one-way ANOVA, F(2,10) = 3.34, *p* = 0.077). Na_2_S_2_O_8_ was therefore selected for the final extraction protocol on the basis of filter cleanliness.

The optimized Na_2_S_2_O_8_ protocol was then applied to larger sample masses in order to enhance organ-level representativeness. For 10 g subsamples of freeze-dried lung tissue, the digestion behaviour closely mirrored that observed for 2 g: filters showed only minor traces of organic residue, filtration proceeded without major difficulties, and microscopic examination remained straightforward. The mean recovery was 69.0 ± 1.4%; in lamb, 10 g of freeze-dried tissue corresponded to ≈25% of the fresh organ represented per subsample. In contrast, when the procedure was applied to 20 g subsamples (≈50% of the fresh lamb lung mass), the method became ineffective. The reagent volume was scaled with respect to the 10 g condition (6 mL/g; 15 mL/g at 2 g), so the reagent-to-tissue ratio at 20 g was lower than at 2 g. Extended digestion times and higher reagent-to-tissue ratios were not tested for 20 g; both remain to be evaluated as ways to increase the mass processed per run. Larger masses were processed in subsamples of up to 10 g. Despite digestion with Na_2_S_2_O_8_, substantial amounts of organic matter persisted, resulting in severely overloaded filters, very long filtration times and, ultimately, the impossibility of reliably analyzing the retained particles, with the mean recovery falling to essentially 0%.

Taken together, Na_2_S_2_O_8_ provided the most efficient digestion across conditions, consistently yielding the cleanest filters, with recoveries of ≈70% that did not differ significantly from those of the other oxidants, while 10 g of freeze-dried lung tissue represented an optimal compromise between recovery, matrix cleanliness and representativeness. On this basis, lung tissue was subsequently processed in consecutive subsamples of up to 10 g, and the particles detected in each subsample (fibres and fragments) were summed across all subsamples analyzed to obtain the organ-level internal burden of the tissue analyzed (Section 3.2).

**Figure 1:**
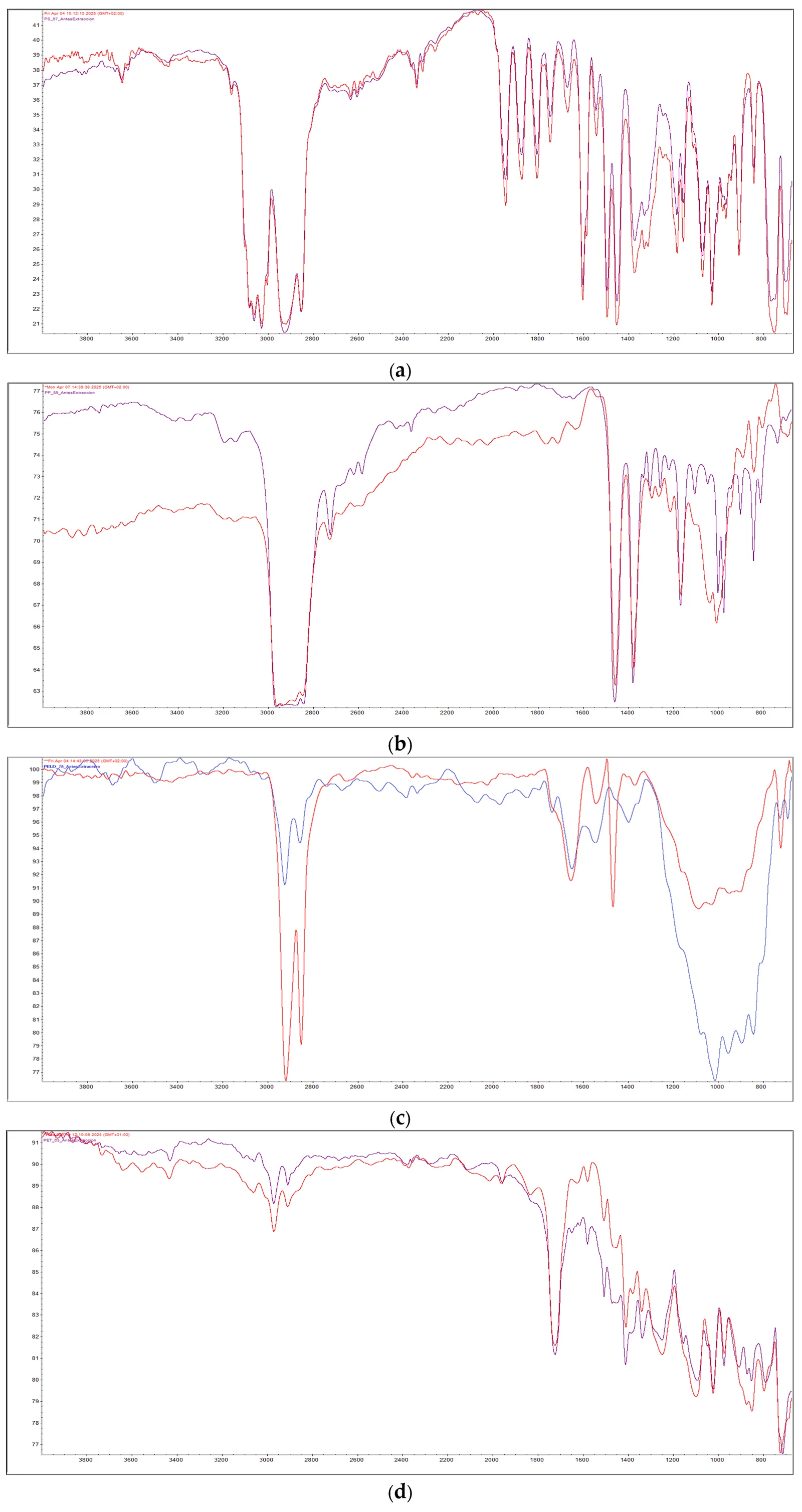
(**a**–**d**) Representative FTIR spectra of pure PS, PP, LDPE, and PET polymers used as reference spectra for microplastic identification. Representative Fourier-transform infrared (FTIR) spectra of pure polystyrene (PS), polypropylene (PP), low-density polyethylene (LDPE) and polyethylene terephthalate (PET) polymers used as reference spectra for microplastic identification. Red: spectrum of the analyzed particle, with acquisition date. Purple (blue in panel (**c**)): reference spectrum from the in-house spectral library. * Spectra processed (blaseline correction). ** Spectra processed (baseline correction and atmospheric suppression).

In Figure 1a–d, representative FTIR spectra are shown for the pure polymers PS, PP, LDPE and PET used in the recovery experiments, together with the corresponding spectra of the four MP types after extraction and analysis from a fortified 10 g lung sample. In each case, the spectrum of the pristine polymer standard (purple line) is compared with that of the recovered MPs following the complete extraction and analytical procedure (red line). In Figure 1a, the characteristic bands of PS are observed. The bands around 3080 cm^−1^ correspond to aromatic C–H stretching, while the signals at 2920 cm^−1^ and 2850 cm^−1^ are associated with aliphatic C–H stretching. The band near 1600 cm^−1^ and those at 1500 and 1450 cm^−1^ are attributed to in-plane and out-of-plane vibrations of aromatic C–H. The similarity between the FTIR spectra before (pristine PS) and after extraction exceeded 90%, confirming the identification of the recovered material as PS. Figure 1b shows the most representative bands for PP. The dominant features appear in the 2950–2850 cm^−1^ region, corresponding to aliphatic C–H stretching vibrations of methyl and methylene groups. Additional bands at 1450–1380 cm^−1^ are attributed to C–H bending modes of methyl groups. In the 1160–980 cm^−1^ region, bands associated with C–C skeletal vibrations are observed. The spectral similarity between the PP standard and the recovered MPs was 84%, which was sufficient to corroborate that the polymer type was PP. In Figure 1c, the characteristic bands of low-density polyethylene (LDPE) are presented. The bands around 2920 and 2850 cm^−1^ are assigned to aliphatic C–H stretching of methylene groups. Bands at 1470 and 1460 cm^−1^ are related to C–H bending vibrations, and additional signals in the 700–750 cm^−1^ region correspond to out-of-plane deformation modes typical of methylene groups. The similarity between the spectra obtained before and after applying the extraction method was greater than 68%, reliably confirming the identity of the analyzed microparticles as LDPE. Figure 1d displays the most representative bands for PET. A distinct band at 1700 cm^−1^ is assigned to the carbonyl (C=O) stretching vibration. Bands at 1250 and 1100 cm^−1^ are attributed to C–O–C stretching, while the band near 1400 cm^−1^ is associated with deformations of the aromatic ring. In the 3100–2800 cm^−1^ region, bands corresponding to aromatic and aliphatic C–H stretching are observed. With a similarity index of 90% between the pristine and recovered spectra, the polymeric nature of the material was unequivocally confirmed as PET.

### 3.2. Internal Burden of Microplastics in Captive-Held Ungulates

The internal burden of MPs was evaluated in three adult gazelles. For Cuvier gazelle and Dorcas 1, a representative portion of each lung was analyzed, made up of small pieces taken from different parts of each lobe (108 and 76 g fresh weight; 21 g and 15 g freeze-dried, respectively); the total lung weights are unknown, so the fraction of the organ analyzed cannot be calculated. The lung of the Dorcas 2 gazelle was analyzed whole (249 g fresh; ≈50 g dry) as five replicates of 10 g. In all cases, the internal burden was obtained as the sum of the particles (fibres and fragments) detected in each of the subsamples analyzed and is expressed per gram of freeze-dried tissue, without correction for recovery. Table 3 gives, for each sample, the fresh weight and dry mass processed, the particles found, the burden per gram of dry tissue (measured and recovery-adjusted) and the detection limit. No synthetic particles were detected in the fetal and neonatal lungs (10 g dry each; detection limit ≈0.14 particles/g dry weight).

In the adult female Dorcas gazelle (Dorcas 1), 4 g of cranial lobe contained nine fibres and three fragments (3 particles/g), 3 g of middle lobe contained 13 fibres and six fragments (6.3 particles/g), and 8 g of caudal lobe contained 24 fibres and 10 fragments (4.25 particles/g). Taken together, the 15 g of freeze-dried tissue analyzed for this animal (76 g fresh weight) contained a total of 65 microplastic particles (46 fibres and 19 fragments), that is to say, 4.33 particles/g.

In the adult female Cuvier’s gazelle, 4 g of cranial lobe contained eight fibres and five fragments (3.25 particles/g), 7 g of middle lobe contained 29 fibres (one with an imputed rather than directly measured width; see Table 4 note) and seven fragments (5.14 particles/g), and 10 g of caudal lobe contained 30 fibres and nine fragments (3.9 particles/g). In this case, the 21 g of freeze-dried tissue analyzed (108 g fresh weight) contained a total of 88 microplastic particles (67 fibres and 21 fragments), that is to say, 4.19 particles/g. In both animals, the highest microplastic burdens per gram were consistently observed in the middle lobe, and fibres clearly predominated over fragments across all lobes.

In the adult female Dorcas gazelle (Dorcas 2), the whole lung (249 g fresh weight; ≈50 g freeze-dried, five replicates of 10 g) contained a total of 27 microplastic particles (19 fibres and eight fragments), that is to say, 0.54 particles/g (replicates: 0.4–0.8 particles/g). This burden per gram was markedly lower than in the other animals; the data do not allow the cause to be identified.

**Figure 2:**
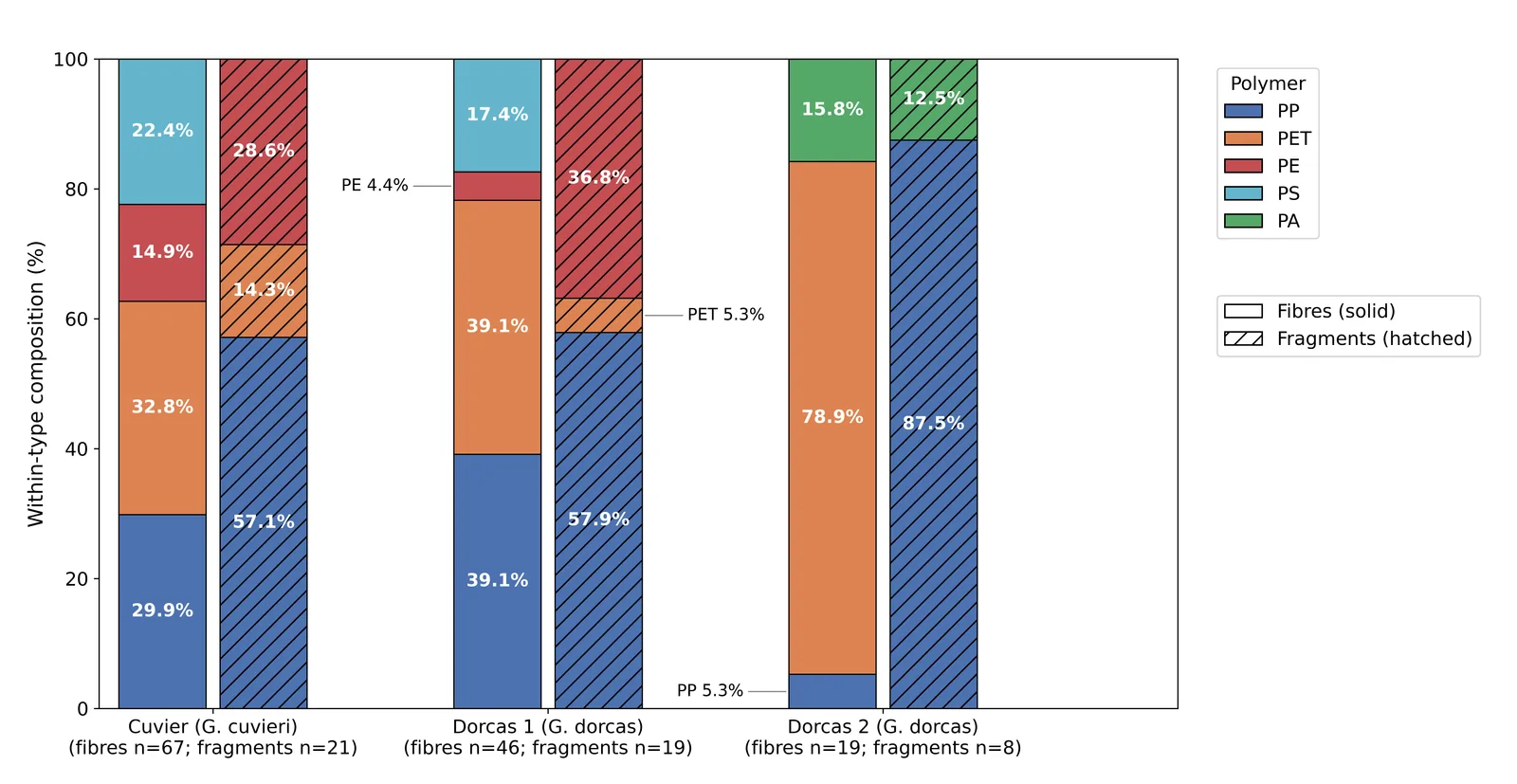
µFTIR-Identified polymer composition by gazelle species and particle type. Polymers: PP, polypropylene; PET, polyethylene terephthalate; PE, polyethylene; PS, polystyrene; PA, polyamide.

Across the three gazelles, fibres were the dominant particle type, representing 70.4–76.1% of all detected MPs (Cuvier: 76.1%; Dorcas 1: 70.8%; Dorcas 2: 70.4%), whereas fragments accounted for 23.9–29.6% (Cuvier: 23.9%; Dorcas 1: 29.2%; Dorcas 2: 29.6%). Regarding polymer composition (Figure 2), a clear contrast emerged between the two particle types: fragments were consistently dominated by PP (Cuvier: 57.1% PP; Dorcas 1: 57.9% PP; Dorcas 2: 87.5% PP), with PE as the secondary component in Cuvier and Dorcas 1 (28.6–36.8%), PA in Dorcas 2 (12.5%), and minor PET where present. For fibres, the distributions were more animal-specific: Cuvier showed a mixed profile (PET 32.8%, PP 29.9%, PS 22.4%, PE 14.9%), Dorcas 1 was dominated by PET and PP (both 39.1%) with a smaller PS contribution (17.4%), while Dorcas 2 was overwhelmingly PET (78.9%) with a notable PA fraction (15.8%) and minor PP (5.3%). Since polymer identity for each particle was confirmed by µFTIR against reference spectral libraries (Section 2.9) rather than inferred indirectly, Figure 2 reflects the measured polymer composition of the fibre and fragment populations analyzed.

**Figure 3:**
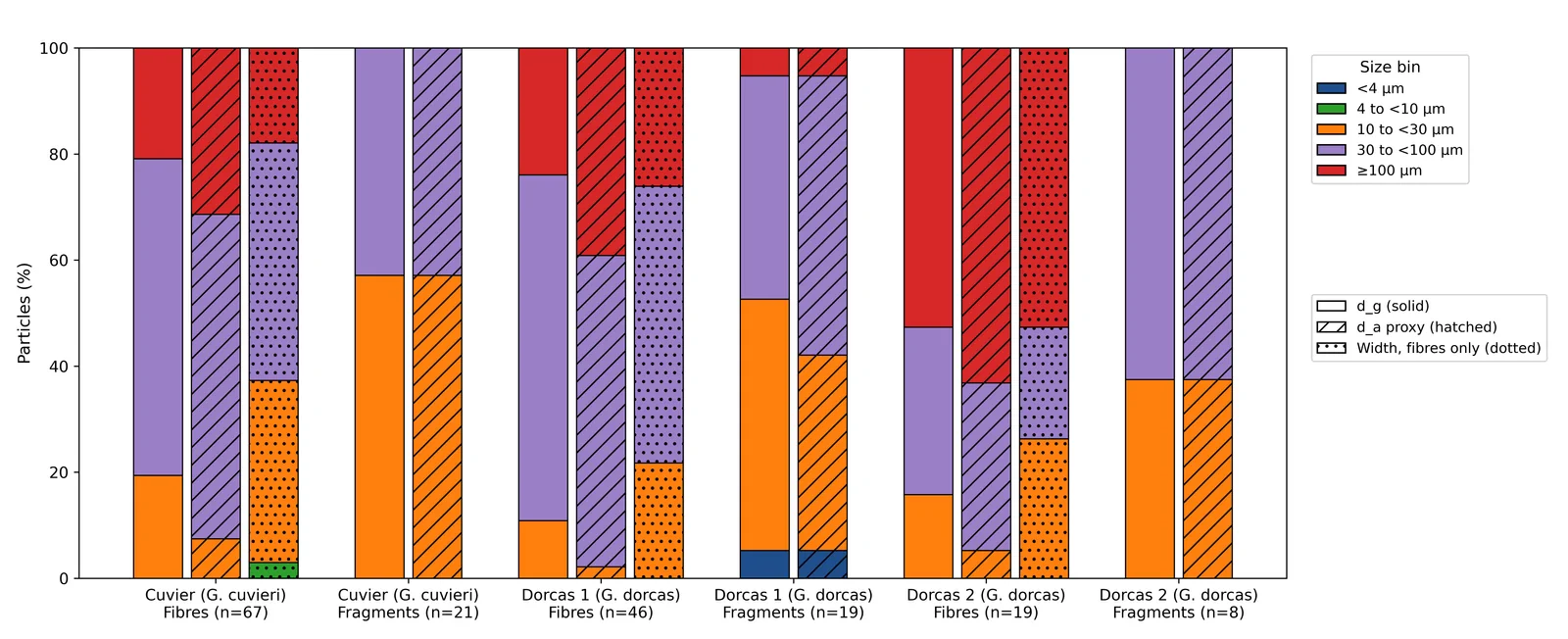
Prevalence by size bins across three gazelles: fibres geometric diameter proxy (d_g) and aerodynamic diameter sensitivity proxy (d_a proxy) and width; fragments (d_g,d_a proxy). For Cuvier fibres (*n* = 67), one fibre with an imputed width is included; Table 4 excludes it from the width-dependent statistics (*n* = 66).

Across the three gazelles, the size distributions are dominated by coarse fibres and medium-to-coarse fragments when expressed as either the geometric proxy (d_g) or the aerodynamic sensitivity proxy (d_a_,proxy). For fibres, most observations fall in the 30 ≤ 100 µm bin (Cuvier ≈ 59–61%; Dorcas 1 ≈ 59–65%; Dorcas 2 ≈ 32%), with a substantial tail in ≥100 µm (Cuvier ≈ 21–32%; Dorcas 1 ≈ 24–39%; Dorcas 2 ≈ 53–63%). Importantly, using d_g or d_a_,proxy, no fibres fall below 10 µm, and none are <4 µm. In contrast, fragments are concentrated in 10 ≤ 30 µm and 30 ≤ 100 µm across animals (typically ~37–63% each), with one single sub-4 µm fragment observed in Dorcas 1 (≈5% of fragments), and essentially no material in the 4 ≤ 10 µm bin. Moving from d_g to d_a_,proxy shifted fibres slightly toward larger apparent sizes (density/shape sensitivity) but did not materially change the classification outcome (i.e., still almost no “thoracic/respirable” particles by the usual cut-offs); only in Cuvier did a small subset of fibres (~3%) show widths <10 µm, and no fibres in any animal were <4 µm by width (see Figure 3).

At the morphometric level, particles recovered from gazelle lungs were dominated by particles with geometric size proxies well above conventional respirable and alveolar ranges, irrespective of animal, lobe, or particle class (Table 4). For fibres, the geometric diameter proxy d_g (defined as √(Width × ECD)) showed median values in the tens to >100 µm, while fragments (for which d_g = ECD) generally exhibited lower medians but still remained predominantly in the coarse micrometre range. The aerodynamic diameter sensitivity proxy d_a (incorporating assumed polymer density and a shape correction) consistently shifted estimated diameters upward relative to d_g, but values remained far above the operational thresholds used for “respirable-like” (<10 µm) and “alveolar-like” (<4 µm) classifications.

In *G. cuvieri*, fibres showed median d_g values of 53.2 µm (caudal), 42.8 µm (middle), and 48.0 µm (cranial), with corresponding d_a proxy medians of 69.3, 50.8, and 65.0 µm, respectively. The 90th percentiles indicated substantial upper tails, particularly in the middle lobe (p90 d_g175.3 µm; p90 d_a211.3 µm). For fragments, median d_g values ranged from 24.8 to 34.9 µm across lobes, with d_a proxy medians of 29.4–36.3 µm; p90 d_a values remained below ~75 µm. Under both d_g and d_a-based screening, no fibres or fragments fell below 10 µm or 4 µm in any lobe (0% “respirable-like” and 0% “alveolar-like” for all categories). In Dorcas (gazelle 1), fibres were consistently large, with median d_g values of 57.3 µm (caudal), 78.0 µm (middle), and 49.4 µm (cranial), and d_a proxy medians of 73.8, 98.0, and 72.6 µm. The p90 values for fibres were again high (p90 d_a~175–181 µm). Fragments in Dorcas 1 displayed greater inter-lobe variability: caudal and middle lobes showed medians of 24.7–30.6 µm (d_a proxy medians 26.2–32.0 µm), whereas the cranial lobe fragments were markedly larger (median d_g 66.8 µm; median d_a 70.8 µm) (*n* = 3). Notably, Dorcas 1 was the only dataset in which any particles met the operational “respirable-like” and “alveolar-like” cut-offs: in the caudal lobe, 10% of fragments (1 out of 10) were <10 µm and <4 µm by both d_g and d_a proxy, while all fibres in this animal remained above both cut-offs.

Dorcas (gazelle 2), analyzed as whole lung, exhibited the largest fibre sizes among the three gazelles. The fibre median d_gwas 101.2 µm with a d_aproxy median of 145.4 µm and a p90 d_a of 362.5 µm; fragments showed median d_gand d_aproxy values of 37.8 µm and 40.3 µm, respectively, and no fibres or fragments fell below 10 µm or 4 µm by either criterion.

### 3.3. Absence of Detectable Microplastics in Fetal and Neonatal Lung Tissue

In marked contrast to the consistent microplastic burdens recovered from the three adult gazelles (Section 3.1 and Section 3.2), no microplastic particles were detected—either by stereomicroscopic screening or by µFTIR confirmation—in the lung tissue of either fetus (male or female) from the pregnant Cuvier’s gazelle, nor in the lung of the neonatal aoudad (*Ammotragus lervia*; Table 1). These samples were collected, stored, digested and analyzed following exactly the same necropsy protocol, QA/QC measures, contamination controls and µFTIR workflow (Section 2.1, Section 2.2, Section 2.3, Section 2.4, Section 2.5, Section 2.6, Section 2.7, Section 2.8 and Section 2.9) applied to the adult specimens in which microplastics were consistently identified, and procedural blanks processed in parallel remained clean throughout.

## 4. Discussion

### 4.1. Method Performance and Comparison with Previous Digestion Strategies

The evaluation of sample mass and oxidizing conditions was designed to maximize MP extraction from lung tissue while avoiding the compromise of particle morphology for microscopic characterization or polymer integrity for FTIR identification, with the ultimate goal of reducing the organic load to a level compatible with reliable analysis without sacrificing recovery. Viewed in this light, the very low recovery observed in the absence of any oxidant (~30%) indicates that extraction efficiency under these conditions was clearly inadequate, while the recovery obtained with Fenton’s reagent (69.4 ± 3.8%) falls within acceptable ranges by typical method validation criteria. The recovery achieved with KOH (63.8 ± 4.3%) was slightly lower than that obtained with Fenton’s reagent (69.4 ± 3.8%) and with Na_2_S_2_O_8_ (70.5 ± 4.1%), but the three did not differ significantly (one-way ANOVA, F(2,10) = 3.34, *p* = 0.077); moreover, in the KOH treatment, the particles were separated with NaCl instead of CaCl_2_, so oxidant and density medium are confounded. Na_2_S_2_O_8_ was selected because it was accompanied by markedly superior matrix cleanliness. Likewise, the maintained recovery at 10 g (69.0 ± 1.4%) confirms that the method could be scaled up in terms of sample mass without loss of performance, while its complete failure at 20 g (recovery 1.0 ± 2.2%), led it to be deemed unsuitable for routine application. Extended digestion times and higher reagent-to-tissue ratios were not tested for 20 g; both remain to be evaluated as ways to increase the mass processed per run. Taken as a whole, these results show that the use of an oxidant and the sample mass are critical determinants of method performance, providing a robust basis for the subsequent application of the method to ungulate lungs.

This aspect is particularly relevant in the broader context of MP analysis in biological tissues. Since the first evidence of MPs in human placenta was reported in 2021 [4], a rapidly growing body of work has focused on detecting MPs in a wide range of human and animal organs, tissues and fluids [36]. In these studies, different sample treatment strategies have been used with the overarching objective of removing organic matter without damaging the polymers present. Alkaline chemical digestion with KOH and oxidative digestion with hydrogen peroxide (H_2_O_2_) or Fenton’s reagent have become the most common approaches, as they are generally effective at solubilizing proteins and lipids while preserving the integrity of most MPs, and are particularly attractive for tissues with a high organic load [36,37]. Our optimization results in lamb lung are consistent with this general picture, but also highlight important limitations of these conventional reagents when applied to heavily vascularized, lipid- and protein-rich tissues such as lung. In our study, KOH digestion produced relatively clean filters and facilitated microscopic examination, but recoveries were somewhat lower (≈64%, not significantly different from the other oxidants), and NaCl instead of CaCl_2_ was used for density separation, so the effect of the oxidant cannot be separated from that of the medium. Fenton’s reagent achieved slightly higher recoveries (≈69%), in line with its widespread use in the literature, yet residual organic matter on the filters continued to hinder visual inspection and microplastic characterization. These findings underscore the trade-off often reported in previous work: reagents that are sufficiently aggressive to digest dense biological matrices tend to improve cleanliness but may also increase the risk of particle loss or polymer alteration, whereas milder treatments can leave behind substantial organic residues that compromise counting and identification [36,37]. In this context, the performance of Na_2_S_2_O_8_ in our validation experiments is particularly noteworthy. Under the conditions applied in this study, Na_2_S_2_O_8_ provided more efficient degradation of lung tissue than KOH or Fenton’s reagent, yielding markedly cleaner filters with recoveries comparable to those of the other oxidants (≈70%) across the test conditions. This is especially relevant for lung, where the combination of blood, lipids and connective tissue presents a more challenging matrix than others such as muscle or gastrointestinal tissues. The superior balance between matrix removal, MP preservation and FTIR-identification reliability achieved with Na_2_S_2_O_8_ suggests that persulfate-based digestion may be a valuable alternative to the classic KOH/H_2_O_2_/Fenton schemes for this type of tissue, if conditions are carefully controlled to avoid polymer degradation.

Previous studies have also explored other digestion approaches, including strong mineral acids such as HNO_3_ and enzymatic protocols based on Proteinase K or mixed enzyme preparations (e.g., Colorase 7089) [37]. Although HNO_3_ can provide very effective digestion, its corrosive nature and documented capacity to degrade certain polymers make it less suitable for quantitative MP studies. In contrast, enzymatic digestion offers high efficiency and specificity with minimal impact on plastic integrity, but requires careful optimization of enzyme concentration, cost considerations and longer processing times [37]. In most published protocols, chemical or enzymatic digestion is combined with density separation using saline solutions such as NaCl, ZnCl_2_ or NaI to isolate MPs from mineral residues [38,39]. PET (1.33–1.45 g/cm^3^ [35]) overlaps with the theoretical density of saturated CaCl_2_ (≈1.40 g/cm^3^), so part of the PET would be expected to float and part to sink. Spiked PET fibres were nevertheless recovered at 62.0–68.0% with Na_2_S_2_O_8_ (see Table 2) and also at 52.5 ± 12.6% when NaCl (≈1.2 g/cm^3^) was used with KOH, which indicates that recovery was not governed by flotation alone. Possible explanations include incomplete sedimentation, adhesion to floating residues or bubbles, aggregation with residual lipids and the way the supernatant was collected; the pellet was not examined, and none of these possibilities was tested. Recovery of larger PET fibres, such as those found in the lungs, and of other dense polymers may therefore differ from that measured. Higher density media such as ZnCl_2_ or NaI, and analysis of the pellet, should be evaluated in future work. Our approach, based on Na_2_S_2_O_8_ digestion followed by CaCl_2_ (and, where necessary, NaCl) density separation, fits within this general framework but is tailored to the specific challenges of lung tissue.

Overall, the comparison with previous digestion strategies indicates that there is no universally optimal reagent for all biological matrices, and that method development must be matrix-specific. For lung tissue, our results show that Na_2_S_2_O_8_ offers a robust compromise between efficient removal of organic matter, acceptable recoveries, preservation of polymer integrity for FTIR identification and practical applicability at sample masses large enough (10 g freeze-dried, ≈25% of the organ) to support organ-level burden estimation. This provides an advance over many existing studies that do not report recovery efficiencies for the extraction step and therefore cannot quantify internal MP burdens at the organ scale. Counts were not corrected for recovery because it was determined with pristine particles of four polymers (PS, PP and LDPE fragments; PET fibres < 50 µm) in lamb lung and may differ for the particles and tissue of the gazelles. Recovery varied among polymers (62.0–72.0% at 10 g), and PA, present in the gazelles, was not tested; the adjusted values were therefore bracketed with the lowest and highest polymer recoveries.

Consistent with this, the spectral comparisons shown in Figure 1 demonstrate that the Na_2_S_2_O_8_- based extraction protocol does not compromise polymer identification by FTIR for any of the MP types studied. The high similarity indices obtained between pristine standards and recovered particles for PS, PP, LDPE and PET, together with the preservation of all key diagnostic bands in their characteristic spectral regions, indicate that the oxidative digestion conditions were sufficiently strong to remove the organic matrix while remaining mild enough to avoid detectable polymer oxidation or spectral distortion. The method therefore ensures both efficient matrix removal and reliable polymer type assignment for MPs isolated from lung tissue.

### 4.2. Particle Type, Polymer Composition and Likely Sources

The predominance of fibres over fragments observed across the three gazelles is consistent with an exposure scenario dominated by airborne fibrous materials, which are more readily transported and retained in the respiratory tract than compact particles, mainly through interception and retention within the conducting airways, given the coarse fibre widths measured here (Section 3.2). The recurring PP enrichment observed among fragments, meanwhile, is compatible with the high prevalence of PP in common consumer and packaging materials and its propensity to generate brittle debris upon weathering, whereas the more animal-specific fibre polymer profiles suggest heterogeneous source contributions, such as synthetic textiles or technical polymers, and potentially different microenvironments or exposure histories among individuals. Even so, the clear separation between a PP-rich fragment signature and a PET/PA-rich fibre signature in some animals is useful for framing hypotheses on sources and transport pathways, and it supports the broader conclusion that pulmonary microplastic burdens in these gazelles are primarily fibre- driven, with fragments forming a consistent secondary component.

The animals were kept in captivity at the La Hoya Experimental Farm in Almería, Spain, and were born in captivity. Because the animals were captive-held, the enclosure environment is a potential source of the particles detected. The fences, shelters and feeders were metallic, and the ground consisted of sand and concrete. Therefore, the main plastic-containing items inside the enclosures were the salt-lick holders and the shade cloth of the sunshades, which could release fibres or fragments through weathering under the strong solar radiation and wind of the area. Windblown plastic bags also occasionally reach the enclosures. Feed and its packaging may represent additional potential sources of microplastic exposure, while feed dust may constitute a potential route of inhalation exposure. Fibre dominance is also compatible with airborne textile-derived fibres and dust originating inside or outside the facility. However, as no environmental samples (air, dust or feed) were analyzed, the contribution of these potential sources to the particles detected cannot be established, and the burdens reported reflect exposure in this captive setting and should not be extrapolated to free-living populations.

### 4.3. Deposition Patterns and Respirability of the Recovered Particles

The consistently higher microplastic burdens observed in the middle lobe of both animals suggest a possible preferential deposition pattern in this region, although the limited number of individuals precludes firm anatomical or mechanistic conclusions. For Cuvier gazelle and Dorcas 1, the burden refers to a representative portion of the lung made up of small pieces from each lobe. Heterogeneity within a lobe is partly averaged, but each lobe contributes to the pooled value in proportion to the mass sampled from it (the middle lobe, with the highest density, contributed 33.3% of the tissue analyzed in Cuvier and 20.0% in Dorcas 1), so the representativeness for the whole lung cannot be verified. Relative to the mean of the tissue analyzed, the density of a single lobe has under- or overestimated the burden by −31.0% to +46.0% (Dorcas 1) and by −22% to +23% (Cuvier). The predominance of fibres over fragments across all lobes is, in turn, in line with the efficient capture of elongated particles, which tend to align with airflow and are readily intercepted at airway walls and bifurcations; given the coarse fibre widths measured here, this capture is expected to occur predominantly within the conducting airways rather than in the distal, gas-exchange region. Nevertheless, the consistent presence of fragments indicates that more isotropic, compact morphologies can also be inhaled and retained across all sampled lobes and should therefore not be neglected in exposure and risk evaluations.

From an inhalation/deposition perspective, these data support a scenario where most detected particles are inhalable but not respirable under conventional aerosol conventions: particles/fibres in the coarse range (~10–100 µm) are preferentially deposited by inertial impaction and interception in the upper airways and conducting (tracheobronchial) region, whereas alveolar deposition is typically associated with much smaller aerodynamic diameters (commonly operationalized as <4 µm) and is therefore not strongly supported by the present size proxies. For fibres, the most physiologically relevant “critical dimension” for penetration is the width (or curve width) rather than length; applying that logic is consistent with the very low fraction of “thoracic-like” fibres: only Cuvier shows a small subset with width <10 µm (~3%), and no fibres are <4 µm by width, indicating that deep-lung/alveolar access—if it occurs—would be limited to a very small subset and/or driven by orientation effects not captured in 2D morphometry.

A further point warrants explicit consideration when interpreting fibre-related size thresholds. Because fibre penetration into the respiratory tract is governed primarily by the cross-sectional (width) dimension rather than by overall length, a fibre with a width at or below the ~4 µm alveolar-like cut-off can, in principle, be markedly longer—often by one to two orders of magnitude, consistent with the fibre length distributions summarized in Table 4 (medians of roughly 40–100+ µm)—without this greater length necessarily precluding deep airway penetration. This diameter-governed, largely length-independent behaviour mirrors long-established fibre-toxicology and fibre-transport paradigms developed for other durable, elongated inhaled particulates (e.g., asbestos and other mineral fibres), in which fibres combining a small diameter with substantial length are of particular concern: they can reach distal airways yet, owing to their length, resist complete engulfment and clearance by alveolar macrophages (“frustrated phagocytosis”), favouring biopersistence and localized retention rather than removal [40,41,42,43,44]. In the present dataset, fibres narrower than 10 µm were found only in the Cuvier’s gazelle (2 of 66 fibres with a measured width), and no fibres narrower than 4 µm were detected. However, since particles below ≈10 µm were outside the size range covered by the method (Section 2.9), the absence of thinner fibres cannot be confirmed, and this scenario can neither be supported nor ruled out with the present data. It nonetheless implies that diameter-resolved reporting of fibre dimensions—as already applied here through width-based screening—should be prioritized over combined, area-inclusive metrics (such as d_g, which scales with both width and projected area, and therefore increases with length) in future microplastic-fibre studies, since the latter can under-flag thin, long fibres of potential toxicological relevance that a width-only criterion correctly identifies as respirable- or alveolar-like.

The detection of fibres and fragments in middle/caudal lobes is still biologically plausible without requiring a predominantly alveolar-sized aerosol. In quadrupeds, regional ventilation patterns, airway branching, and gravitational effects can favour deposition in the middle and caudal lobes for certain particle types, and retention/redistribution mechanisms (mucociliary transport, mechanical lodging at bifurcations, and “frustrated phagocytosis” for long fibres) can contribute to their persistence within the airways of these lobes even when the particles are not classically “respirable.” In short, the size distributions provide strong evidence of exposure and retention dominated by coarse, airway-interacting MPs, with minimal support for an alveolar-sized burden based on d_g and d_a_,proxy, and width-based screening.

At the individual level, the broad upper tail observed in the middle lobe of *G. cuvieri* (p90 d_g 175.3 µm; p90 d_a 211.3 µm) suggests a broader size distribution and the presence of very large fibres in that region, and collectively the data from this animal indicate that its particles are overwhelmingly non-respirable under standard aerosol conventions, meaning that any interpretation of deep-lung penetration must rely on anatomical localization rather than size-based expectations alone. In Dorcas 1, the similarly high p90 values for fibres (~175–181 µm) are consistent with frequent occurrence of thick and/or high-drag fibres, while the larger fragment estimates in the cranial lobe should be interpreted cautiously given the small underlying sample (*n* = 3), which increases sensitivity to outliers and sampling variance. The single fine fragment identified in this animal’s caudal lobe, meeting both the <10 µm and <4 µm cut-offs, suggests the presence of a rare fine fragment capable, in principle, of deeper penetration under aerosol criteria; however, because the overwhelming majority of particles in Dorcas 1 remained far above 10 µm and none of the fibres in this animal fell below either cut-off (0%), the key biological inference remains dominated by large particles, and this single fine fragment should be interpreted cautiously and in light of analytical blanks, segmentation confidence, and spatial localization in tissue. In Dorcas 2, the markedly large fibre median and p90 values point to a substantial fraction of extremely large fibres, and the fragment medians, again well above the respirable range, reinforce the broader pattern that, while fragments are generally smaller than fibres, both particle classes are dominated by sizes that would typically deposit in upper or conducting airways rather than the alveolar region if interpreted strictly through aerosol cut-offs.

Taken together, the near-complete absence of particles with d_g or d_a,proxy below 10 µm across all three gazelles, and the singular occurrence of one <4 µm fragment, indicates that the microplastics detected are predominantly in a size regime not typically associated with alveolar deposition under standard human-oriented aerosol definitions. It is worth noting that these thresholds are operational and derived from human exposure science; while they provide a useful comparative framework, they cannot be assumed to map directly onto gazelle airway geometry and breathing patterns. Nevertheless, the conclusion appears robust to reasonable shifts in cut-offs, because median d_g and d_a,proxy values for fibres and fragments remain far above 10 µm in all animals and regions. The d_a,proxy should be viewed as a sensitivity analysis rather than a direct aerodynamic measurement, since it depends on assumed polymer compositions/densities and a shape correction inferred from 2D morphometry. However, even the purely geometric screening using d_g yields the same qualitative outcome.

The detection of large fibres and fragments in lobe-resolved lung samples therefore likely reflects mechanisms other than classical “respirable-to-alveolar” transport. For fibres in particular, deposition by interception at airway bifurcations, entrapment in mucus within the tracheobronchial tree, and retention driven by local airway architecture can lead to regional accumulation in sampled lobes without requiring true alveolar penetration. Consequently, the most defensible inference from Table 4 is that the measured particle populations are consistent with deposition and/or retention predominantly within conducting airways (bronchial/bronchiolar compartments), with limited evidence for an appreciable alveolar-sized fraction. Where claims regarding alveolar localization are desired, they should be supported by histological confirmation (e.g., parenchymal localization versus airway lumen/mucus) rather than size-based classification alone.

### 4.4. Absence of Microplastics in Fetal and Neonatal Lung Tissue

Because the fetal and neonatal samples were processed following exactly the same protocol, QA/QC measures and contamination controls as the adult specimens, and because procedural blanks run in parallel remained clean, these negative results are unlikely to reflect a failure of the analytical method itself. Several, non-mutually exclusive explanations may account for this pattern. Fetal lungs remain fluid-filled and non-ventilated in utero, so any fetal exposure to airborne MPs could only occur indirectly, via maternal inhalation followed by systemic absorption and transplacental transfer—a route documented for MPs in human placenta and increasingly discussed for other fetal tissues [4,7,33,34]. The absence of detectable particles in either fetus is therefore compatible with a genuinely low or negligible transplacental transfer of MPs to the fetal lung in this particular pregnancy, or with a true barrier effect at the placental interface for the particle types and sizes prevailing in the dam’s lung. Separately, the neonatal aoudad had accumulated only a very short period of postnatal respiratory exposure at the time of death, in sharp contrast with the adult gazelles, whose pulmonary burden reflects years of cumulative inhalation. Because the particles identified in adult lungs were predominantly coarse fibres and fragments consistent with mechanical retention in conducting airways rather than rapid mucociliary clearance (Section 4.3), pulmonary MP burden in these ungulates plausibly behaves as a slowly accumulating, largely irreversible deposit; under this hypothesis, a neonate would not yet be expected to have accumulated a burden large enough to be captured by whole-lung digestion, even though the same protocol reliably detects such burdens in adults. This is consistent with an age-dependent, cumulative-exposure model for pulmonary MPs, analogous to patterns described for other durable, poorly biodegradable inhaled particulates, and points toward postnatal, airway-mediated deposition—rather than prenatal, transplacental transfer—as the principal route by which the fibre- and fragment-dominated burden described in Section 3.2 is established.

From a quality-assurance standpoint, these negative results also carry methodological value: because the fetal and neonatal samples were exposed to exactly the same handling and laboratory environment, and therefore to the same contamination risk, as the adult lungs in which MPs were readily and reproducibly detected, their negative outcome provides indirect, reassuring evidence against contamination as the source of the particles reported in the adults—had cross-contamination been a major contributor, a comparable background would be expected irrespective of the donor’s respiratory history. We nonetheless stress that, with a single pregnancy and a single neonate examined, these negative findings are hypothesis-generating rather than conclusive, and that adequately powered studies pairing maternal, fetal and neonatal tissues—ideally combined with maternal blood or placental analysis—will be required to formally test prenatal transfer and to characterize the postnatal time-course of pulmonary MP accumulation.

## 5. Conclusions

This study establishes a validated analytical framework for quantifying microplastics in lung tissue that, unlike previous work on animal or human organs, explicitly incorporates extraction recovery efficiency together with organ-level, rather than subsample-only, burden estimation. Na_2_S_2_O_8_-based oxidative digestion of 10 g freeze-dried tissue offered the best compromise between matrix removal, particle and polymer preservation, and practical scalability, and this protocol proved transferable, without modification, to lung tissue from captive Saharan gazelles. Applying the method to two adult gazelles and to a whole lung from a third individual revealed a consistent, fibre-dominated internal burden with µFTIR-identified polymer signatures compatible with textile- and packaging-derived sources, and size distributions that place the great majority of detected particles above conventional respirable and alveolar cut-offs. Because transmission µFTIR is diffraction-limited at wavelengths comparable to the ~4 µm alveolar-like threshold used here, and because thin fibres can combine a small, potentially respirable diameter with a much greater length, these size-based conclusions should be regarded as conservative rather than exhaustive, and complementary techniques offering finer spatial resolution (e.g., µRaman) are recommended to extend size coverage below 4 µm in future work. These findings suggest that inhalation exposure and retention of microplastics in these ungulates predominantly involve conducting-airway rather than alveolar deposition, although the operational thresholds used here are derived from human aerosol science and should be interpreted cautiously when extrapolated to quadruped airway anatomy. Notably, no microplastics were detected in either fetus or in the neonatal aoudad processed under the identical protocol, a negative result that both reinforces confidence that the burdens measured in adult lungs are not laboratory artefacts and points toward postnatal, airway-mediated accumulation—rather than substantial prenatal transplacental transfer—as the principal route establishing pulmonary MP burden in these ungulates, a hypothesis that larger, purpose-designed studies pairing maternal, fetal and neonatal tissue should now test directly. Given its recovery-characterized, organ-level design, this method provides a reproducible basis for comparative studies of respiratory MP exposure across mammalian species, including its use as a bioindicator approach in captive-held life and livestock health monitoring.

## Figures and Tables

**Table 1:** Characteristics of ungulates undergoing necropsy for microplastic (MPs) evaluation in lung tissue: species, age, sex, and cause of death.

Species	Animal	Sex	Date of Birth	Date of Death	Age at Death	Date of Necropsy	Cause of Death
Cuvier’s gazelle	1440	Female	21 April 2012	30 April 2024	12 years	3 May 2024	Pregnancy toxemia
Cuvier’s gazelle	Fetus	Female	Unborn	30 April 2024	—	3 May 2024	Intrauterine death
Cuvier’s gazelle	Fetus	Male	Unborn	30 April 2024	—	3 May 2024	Maternal death
Dorcas gazelle	1544	Female	15 October 2012	12 May 2024	11 years	16 May 2024	Advanced age
Dorcas gazelle	1371	Female	17 August 2009	24 June 2025	15 years	26 June 2025	Rumen sarcoma
Saharan aoudad	546	Female	4 August 2025	9 August 2025	5 days	12 August 2025	Starvation due to maternal neglect

A long dash means 0 years, as there not born.

**Table 2:** Polymer-specific and total recovery rates (mean ± SD,%) of spiked microplastics (PS, PP, LDPE, PET; 10 particles per polymer) under different digestion conditions. Raw counts per replicate are given in Table S1.

Condition	*n* Analyzed	PS	PP	LDPE	PET	Total (SD; %RSD)
Na_2_S_2_O_8_, 2 g	5	74.0 ± 5.5	68.0 ± 8.4	72.0 ± 11.0	68.0 ± 8.4	70.5 ± 4.1 (5.8%)
Na_2_S_2_O_8_, 10 g	5	72.0 ± 8.4	72.0 ± 4.5	70 ± 7.1	62.0 ± 4.5	69.0 ± 1.4 (2.0%)
Na_2_S_2_O_8_, 20 g	5	2.0 ± 4.5	2.0 ± 4.5	0.0 ± 0.0	0.0 ± 0.0	1.0 ± 2.2 (224%)
Fenton, 2 g	4	72.5 ± 15.0	70.0 ± 0.0	70.0 ± 8.2	65.0 ± 10.0	69.4 ± 3.8 (5.4%)
KOH + NaCl	4	70.0 ± 0.0	55.0 ± 5.8	77.5 ± 5.0	52.5 ± 12.6	63.8 ± 4.3 (6.8%)
CaCl_2_, 2 g	2	25.0 ± 7.1	15.0 ± 21.2	45.0 ± 35.4	35.0 ± 35.4	30.0 ± 10.6 (35.0%)

SD, Standard deviation; RSD, relative standard deviation; PS, polystyrene; PP, polypropylene; LDPE, low-density polyethylene; PET, polyethylene terephathalate; KOH, potassium hydroxide; NaCl, sodium chloride; CaCl_2_, calcium chloride; Na_2_S_2_O_8_ sodium persulfate.

**Table 3:** Microplastic abundance, morphological distribution (fibres and fragments), and recovery-adjusted concentrations in individual tissue samples, including processed sample weights and detection limits. Recovery-adjusted values are measured values divided by 0.69 (mean recovery at 10 g); ranges in parentheses use the lowest (62.0%) and highest (72.0%) polymer-specific recoveries at 10 g.

Sample	Lobes	Fresh Weight (g)	Total Fresh Weight (g)	Dry Mass (g)	Total Dry Mass (g)	Fibres	Fragments	Total Particles	Particles/g Dry	Recovery- Adjusted Particles/g Dry	Detection Limits (Particles/g Dry)
Cuvier	Cranial	22	108	4	21	67	21	88	4.19	6.1 (5.8–6.8)	0.07
	Middle	36		7							
	Caudal	50		10							
Dorcas 1	Cranial	21	76	4	15	46	19	65	4.33	6.3 (6.0–7.0)	0.1
	Middle	14		3							
	Caudal	41		8							
Dorcas 2	Whole lung		249		≈50.0	19	8	27	0.54 ± 0.017	0.8 (0.75–0.87)	0.03 (whole lung); 0.14 per 10 g run
Fetus 1 (Cuvier)	Whole lung		51		≈10.2	0	0	0	no synthetic particles found	-	0.14
Fetus 2 (Cuvier)	Whole lung		52		≈10.4	0	0	0	no synthetic particles found	-	0.14
Neonate (aoudad)	Whole lung		51		≈10.2	0	0	0	no synthetic particles found	-	0.14

**Table 4:** Aerodynamic diameter proxy (d_a), geometric diameter (d_g), and respirability classification by lobe. *Gazella cuvieri* (Cuvier’s gazelle) and *Gazella dorcas* (Dorcas gazelle). Respirable: <10 µm; alveolar-like: <4 µm. d_a is a sensitivity proxy (not a direct measurement).

Gazelle	Type	Lobe/Region	*n*	d_g Definition	Median Width (µm)	Width p90 (µm)	Minimun Width (µm)	% < 10 µm (*n*)	% < 4 µm (*n*)	d_g Median (µm)	d_g p90 (µm)	d_a Proxy Median (µm)	d_a Proxy p90 (µm)	Respirable (% < 10 µm) by d_g	Alveolar-like (% < 4 µm) by d_g	Respirable (% < 10 µm) by d_a	Alveolar-like (% < 4 µm) by d_a
Cuvier (*G. cuvieri*)	Fibres	caudal	30	sqrt(Width × ECD)	50.2	113.6	9.6	3.3 (1)	0	53.2	111.8	69.3	151.9	0	0	0	0
Cuvier (*G. cuvieri*)	Fibres	middle	28	sqrt(Width × ECD)	33.0	228.3	8.9	3.6 (1)	0	42.8	175.3	50.8	211.3	0	0	0	0
Cuvier (*G. cuvieri*)	Fibres	cranial	8	sqrt(Width × ECD)	36.9	144.5	12.8	0	0	48	123.3	65	186.8	0	0	0	0
Cuvier (*G. cuvieri*)	Fragments	caudal	9	ECD	-	-	-	0	0	34.9	64.7	36.3	73.1	0	0	0	0
Cuvier (*G. cuvieri*)	Fragments	middle	7	ECD	-	-	-	0	0	29.0	42.2	29.4	59.1	0	0	0	0
Cuvier (*G. cuvieri*)	Fragments	cranial	5	ECD	-	-	-	0	0	24.8	52	29.7	59.1	0	0	0	0
Dorcas 1 (*G. dorcas*)	Fibres	caudal	24	sqrt(Width × ECD)	50.4	155.1	15.3	0	0	57.3	142.9	73.8	175.2	0	0	0	0
Dorcas 1 (*G. dorcas*)	Fibres	middle	13	sqrt(Width × ECD)	71.2	167.9	13.8	0	0	78	143.9	98	175.1	0	0	0	0
Dorcas 1 (*G. dorcas*)	Fibres	cranial	9	sqrt(Width × ECD)	43.0	168.5	22.5	0	0	49.4	137.1	72.6	180.9	0	0	0	0
Dorcas 1 (*G. dorcas*)	Fragments	caudal	10	ECD	-	-	-	0	0	24.7	44.7	26.2	48	10	10	10	10
Dorcas 1 (*G. dorcas*)	Fragments	middle	6	ECD	-	-	-	0	0	30.6	59.2	32	65.4	0	0	0	0
Dorcas 1 (*G. dorcas*)	Fragments	cranial	3	ECD	-	-	-	0	0	66.8	170	70.8	195.4	0	0	0	0
Dorcas 2 (*G. dorcas*)	Fibres	full	19	sqrt(Width × ECD)	87.8	270.5	14.1	0	0	101.2	237.8	145.4	362.5	0	0	0	0
Dorcas 2 (*G. dorcas*)	Fragments	full	8	ECD	-	-	-	0	0	37.8	59.7	40.3	62.3	0	0	0	0

Notes: For fibres, d_g = sqrt(Width × ECD). For fragments, d_g = ECD. d_a proxy = d_g × sqrt(particle density) × sqrt(shape correction χ). Polymer densities were assigned according to the polymer type identified for each particle by µFTIR against reference spectral libraries (Section 2.9). For the Cuvier middle lobe, one additional fibre with a positively identified but computationally imputed (rather than directly measured) width was excluded from the width-dependent size statistics shown here (d_g, d_a proxy); it is nonetheless included in the total fibre burden reported in Section 3.2, since its presence as a fibre was unambiguous.

## Data Availability

The data presented in this study are available within the article (Table 1, Table 2, Table 3 and Table 4 and Supplementary Table S1). Additional raw data are available from the corresponding author upon reasonable request.

## Supplementary Materials

The following supporting information can be downloaded at: https://www.mdpi.com/article/10.3390/polym18212786/s1, Table S1: Number of particles recovered out of 10 spiked per polymer.

## Abbreviations

The following abbreviations are used in this manuscript:

MP(s)	Microplastic(s)
KOH	Potassium hydroxide
Na_2_S_2_O_8_	Sodium persulfate
CaCl_2_	Calcium chloride
FTIR	Fourier-Transform Infrared (spectroscopy)
µFTIR	Micro-Fourier-Transform Infrared (spectroscopy)
PS	Polystyrene
PP	Polypropylene
LDPE	Low-Density Polyethylene
PE	Polyethylene
PET	Polyethylene terephthalate
PA	Polyamide
H_2_O_2_	Hydrogen peroxide
HNO_3_	Nitric acid
NaCl	Sodium chloride
ZnCl_2_	Zinc chloride
NaI	Sodium iodide
QA/QC	Quality Assurance/Quality Control
d_g	Geometric diameter (proxy)
d_a (d_a,proxy)	Aerodynamic diameter sensitivity (proxy)
ECD	Equivalent Circular Diameter
p90	90th percentile
