# Supplementary material for: Respiratory Exposure to Microplastics in Captive-Held Mammals: Validated Lung Tissue Method with Recovery Efficiency and Internal Burden Evaluation in Ungulates
Source: Polymers (Basel). 2026 Nov 7;18(21):2786. doi: 10.3390/polym18212786 (PMC13765667; doi:10.3390/polym18212786)
Supplement: Supplementary file 1 [file polymers-18-02786-s001.zip › polymers-4645968-supplementary.pdf]

Table S1. *Number of particles recovered out of 10 spiked per polymer. The total number of particles spiked per replicate was 40.*

Treatment	Replicate	PS	PP	LDPE	PET	Total recovered
	R1	8	6	9	6	29
	R2	7	8	7	8	30
$Na_2S_2O_8$, 2 g	R3	7	6	7	7	27
	R4	7	7	6	6	26
	R5	8	7	7	7	29
	R1	7	7	7	6	27
	R2	6	7	8	7	28
$Na_2S_2O_8$, 10 g	R3	7	8	6	6	27
	R4	8	7	7	6	28
	R5	8	7	7	6	28
	R1	1	1	0	0	2
	R2	0	0	0	0	0
$Na_2S_2O_8$, 20 g	R3	0	0	0	0	0
	R4	0	0	0	0	0
	R5	0	0	0	0	0
	R1	-	-	-	-	The sample was lost
	R2	8	7	6	5	26
Fenton, 2 g	R3	5	7	8	7	27
	R4	8	7	7	7	29
	R5	8	7	7	7	29
	R1	-	-	-	-	Sample loss (precipitate formation)
	R2	7	5	8	4	24
KOH +NaCl	R3	7	6	7	5	25
	R4	7	5	8	5	25
	R5	7	6	8	7	28
	R1	2	0	7	6	15
	R2	3	3	2	1	9
$CaCl_2$, 2 g	R3	-	-	-	-	Not analysable
	R4	-	-	-	-	Not analysable
	R5	-	-	-	-	Not analysable
